# Screening of Polymers for Oral Ritonavir Amorphous Solid Dispersions by Film Casting

**DOI:** 10.3390/pharmaceutics16111373

**Published:** 2024-10-26

**Authors:** Ayse Nur Oktay, James E. Polli

**Affiliations:** 1 Department of Pharmaceutical Technology, Gulhane Faculty of Pharmacy, University of Health Sciences, Ankara 06018, Türkiye; 2 Department of Pharmaceutical Sciences, University of Maryland, Baltimore, MD 21201, USA

**Keywords:** ritonavir, screening, miscibility, polymer, amorphous solid dispersion, film casting

## Abstract

**Background/Objectives:** Drug–polymer interactions and miscibility promote the formation and performance of amorphous solid dispersions (ASDs) of poorly soluble drugs for improved oral bioavailability. The objective of this study was to employ drug–polymer interaction calculations and small-scale experimental characterization to screen polymers for potential ASDs of ritonavir. **Methods:** Seven polymers across four polymer types were screened as follows: an enteric one (EudragitS100), amphiphilic ones (HPMCAS-L, HPMCAS-H, and their 1:1 combination), hydrophilic ones (PEG-6000, PVP-VA), and a surfactant (Soluplus), including PVP-VA as a positive control, as the commercial ASD employs PVP-VA. Drug–polymer interaction calculations were performed for Hansen solubility parameter, Flory–Huggins parameter, and glass transition temperature. ASDs were prepared via film casting. Experimental characterizations included drug solubility in polymer solutions, polymer inhibition of drug precipitation, polarized light microscopy, differential scanning calorimetry, solubilization capacity, and dissolution studies. **Results:** HPMCAS-L, HPMCAS L:H, and Soluplus, along with the positive control PVP-VA, were identified as polymers for potential ASDs of ritonavir, with HPMCAS-L and PVP-VA being preferable. HPMCAS-L and the positive control PVP-VA were always viable for both 20% and 40% drug loads across all tests. Films with each of these four polymers showed improved dissolution compared to amorphous ritonavir without polymer. Drug–polymer interaction calculations anticipated the unfavorable small-scale experimental results for PEG-6000 and EudragitS100. **Conclusion:** Overall, the results contribute towards a resource-sparing approach to identify polymers for ASDs.

## 1. Introduction

There is increasing need for formulations that increase drug solubility [1,2,3]. Diverse approaches such as salt formation; the preparation of inclusion complex, liposome, or solid dispersion formulation; coamorphous systems; and amorphization have been applied to improve aqueous solubility [4]. Several amorphous solid dispersions (ASDs) have been marketed [1,2,5,6,7,8,9].

Amorphous drug–polymer miscibility has been previously described [10,11,12]. Miscibility relates to drug and polymer chemical compositions, as well as t ratio [13,14]. In the selection of polymers for ASDs, polymer properties such as glass transition temperature (T_g_), polymer chemistry (e.g., anionic, cationic), the presence or absence of intermolecular interactions, polymer hygroscopicity, solubility in commonly used organic solvents, polymer molecular weight, and polymer thermal stability should be considered [15,16,17]. Polymers in the preparation of ASDs are often highly soluble in all pH conditions or at least in more alkaline ones. They include hydroxypropylmethyl cellulose (HPMC), hydroxypropylmethyl cellulose phthalate (HPMCP), hydroxypropylmethyl cellulose acetate/succinate (HPMCAS), poly(vinylpyrrolidone) (PVP), poly(vinylpyrrolidone/vinyl acetate copolymer) (PVP-VA), and the acrylic acid-based enteric Eudragit system [18,19].

Methods to prepare amorphous miscible systems include spray drying and hot-melt extrusion. Film casting has been advocated to screen for polymers as a material- and time-sparing technique [4,13,20,21,22,23]. Honick et al. used film casting to prepare itraconazole ASDs with three different types of HPMCAS, as well as different drug loads [13]. Comparison of dissolution profiles of films and spray-dried dispersions (SDDs) supported films in being able to anticipate the polymer and drug load performance of SDDs. Film casting and spray drying are “bottom-up” techniques that rely on the same fundamental process principles (i.e., rapid solvent evaporation). Hence, it may be expected that film casting can provide early development insight into viable drug–polymer pairs for subsequent SDD studies. Mosquera-Girald et al. more recently found film casting, using specific methods, predicted the in vitro and in vivo performance of the corresponding SDDs of three development compounds [24]. However, there continues to be no scientific consensus on the ability of films to predict SDDs, or even the effects of drug load or polymer type.

Using ritonavir (RTN), film casting was assessed here via drug–polymer interaction calculations and small-scale experimental characterization to screen several polymers for potential ritonavir ASDs. Ritonavir was selected since it is commercially available as an ASD, such as in Norvir tablets, using PVP-VA [25]. Seven polymer systems of varying nature, including PVP-VA, were screened here as follows: an enteric one (Eudragit S100) [21,26], amphiphilic ones (HPMCAS-L, HPMCAS-H, and 1:1 combination), hydrophilic ones (PEG-6000, PVP-VA), and a surfactant (Soluplus). PVP-VA served as a positive control in assessing drug–polymer interaction calculations and small-scale experimental characterization to screen polymers, as PVP-VA is a viable polymer for ritonavir ASDs [25]. Drug–polymer interaction and miscibility were estimated via δ_T_, (Hansen total solubility parameter), χ, (Flory–Huggins drug–polymer interaction parameter), and T_g_ prediction via Fox equation [16]. Screening focus comprised 20–40% drug loads, as this range is typical of the literature and marketed products [13,27,28,29,30,31,32,33]. For example, ritonavir release from lower drug loading (i.e., at or below 25%) was rapid, complete, and congruent with polymer release, compared to higher, less viable drug loading (i.e., 40 and 50%) [21,34]. Similar results were observed with lumefantrine ASDs [35].

Films were experimentally characterized by appearance evaluation, differential scanning calorimetry (DSC) analysis, polarized light microscopy (PLM), solubilization capacity determination, and dissolution. The physical stability of films was evaluated after storage for 30 days. Drug–polymer solution-state studies were also performed to assess polymer promotion of drug solubility, as well as polymer inhibition of drug precipitation.

This sequenced approach identified HPMCAS-L and PVP-VA as preferable polymers for subsequent ASD testing, reflecting favorably for this approach to identify promising drug–polymer pairs, as PVP-VA results were optimistic.

Overall, HPMCAS-L and PVP-VA were identified via drug–polymer interaction calculations and small-scale experimental characterization studies of films as the most viable polymers for subsequent ASD assessment, for each of the 20% and 40% drug loads. Soluplus and a 1:1 mixture of HPMCAS L:H also appeared viable for a 20% drug load, while HPMCAS-H, Eudragit S100, and PEG-6000 were not viable. The results will contribute towards an approach to rationally identify polymers for ASDs using a more material- and time-sparing development pathway.

## 2. Materials and Methods

### 2.1. Materials

Polyvinylpyrrolidone-vinyl acetate (PVP-VA, Kollidon^®^VA 64) and polyvinyl caprolactame-polyvinyl acetate + polyethylene glycol graft copolymer (Soluplus) were provided by BASF (Ludwigshafen, Germany). Polyethylene glycol-6000 (PEG-6000) was provided by Affymetrix (Cleveland, OH, USA). Methyl methacrylate copolymer (Eudragit S100) was provided by Evonik (Evonik GmbH, Essen, Germany). Hypromellose acetate succinate L and H grades (HPMCAS-L and HPMCAS-H) were provided by Ashland (Covington, KY, USA). Table 1 lists polymer properties. Polymers were selected to be chemically diverse, as drug/polymer miscibility depends upon drug and polymer chemical structures and their interactions.

Ritonavir was purchased from ChemShuttle (Blue Current Inc., Hayward, CA, USA). Norvir 100 mg tablet (AbbVie, North Chicago, IL, USA) was commercially obtained. Polyoxyethylene (10) lauryl ether (POE10) and solvents were purchased from Sigma Aldrich (St. Louis, MO, USA) and Fischer Scientific (Hampton, NH, USA). Aluminum dishes (57 mm diameter) were purchased from VWR.

### 2.2. Overall Approach

**Figure 1:**
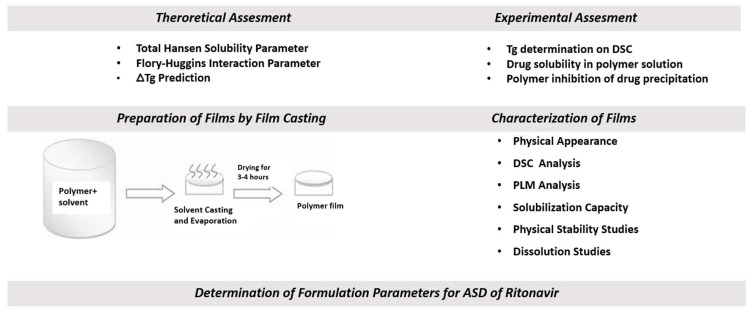
Illustration of scope and sequence of drug–polymer interaction calculations and small-scale experimental characterizations to screen polymers for potential ASDs. This sequenced approach of nine screening test methods, which considered solid- and/or solution-state performance, identified PVP-VA and HPMCAS-L as preferable polymers.

Figure 1 depicts the scope and sequence of drug–polymer interaction calculations and small-scale experimental characterizations performed here. Nine screening test methods were applied. Ritonavir was selected, as it is commercially viable as an ASD with PVP-VA. Seven polymers, including PVP-VA, were assessed though three layers of methods: theoretical calculations, experimental assessment of drug–polymer physical mixtures and solutions, and film characterization. PVP-VA served as a positive control since commercial Norvir tablets employ PVP-VA as the polymer for the ritonavir ASD.

### 2.3. Theoretical Calculations

To predict ritonavir (RTN) miscibility and solubility in polymers, the Hansen total solubility parameter and the Flory–Huggins drug–polymer interaction parameter were calculated. Also, T_g_ was predicted to evaluate amorphous system physical stability.

#### 2.3.1. Total Hansen Solubility Parameter and Flory–Huggins Interaction Parameter

δ_T_ (Hansen total solubility parameter) was calculated for RTN and each polymer by using the Hansen equation:

$$\delta_{T}=\sqrt{{\delta_{d}}^{2}+{\delta_{p}}^{2}+{\delta_{h}}^{2}}.$$

where δ_h_ is the energy from hydrogen bonds between molecules, δ_p_ is the energy from dipolar intermolecular forces between molecules, and δ_d_ is the energy from dispersion forces between molecules. Compound structures were generated using Chemaxon Software Version 5.10.4 (PerkinElmer, Hopkinton, MA, USA); SMILE files of molecules were used as input for the Hansen solubility parameters in practice software version 6.0.04 (HSPiP v6.0.04, London, UK). The solubility parameter was predicted from chemical group contributions of RTN and polymers, using the HSPiP software (version 6.0.04), which is based on the Hoftyzer and Van Krevelen method [16,36].

Also, *χ* (Flory–Huggins drug–polymer interaction parameter) was calculated from the following:

*χ =* (V_site_/RT) × (δ_drug_ − δ_polymer_)^2^

where R is the gas constant (8.31 J·mol^−1^·K^−1^), T is the absolute temperature (298.15 K at 25 °C), and V_site_ is the hypothetical lattice volume (i.e., 581.7 ± 3.0 cm^3^ for ritonavir) [16,36,37]. δ_drug_ and δ_polymer_ were the total solubility parameters of RTN and of polymer, respectively.

#### 2.3.2. Prediction of T_g_ of Amorphous Solid Dispersions

T_g_ (glass transition temperature) of the ritonavir and polymer blend (physical mixture) was calculated for each 20% and 40% drug load using the Fox equation:

$$\frac{1}{T_{g}}=\frac{W_{1}}{T_{g1}}+\frac{W_{2}}{T_{g2}}$$

where *T_g*1*_* and *T_g*2*_* are *T_g_* of the drug and polymer, respectively, and *W_*1*_* and *W_*2*_* are weight fractions of the drug and polymer, respectively. *W_*1*_* = 0.2 and *W_*2*_* = 0.8 for 20% drug-loaded films. *W_*1*_* = 0.4 and *W_*2*_* = 0.6 for 40% drug-loaded films.

### 2.4. Experimental Assessment of Drug–Polymer Physical Mixtures and Solutions

#### 2.4.1. Drug Solubility in Polymer Solution

At two pHs, drug solubility studies were performed with and without 2 mg/mL polymer in buffer. The buffer consisted of 60 mM POE10 in 50 mM maleic acid (pH 5.8 or 6.5). Furthermore, 60 mM POE10 is the USP dissolution medium for ritonavir tablets [38]. Maleic acid buffer was selected since Fed State Simulated Intestinal Fluid Version 2 (FeSSIF-V2) and Fasted State Simulated Intestinal Fluid Version 2 (FaSSIF-V2) employ maleic acid buffer at pH 5.8 and 6.5, respectively. Excess RTN (0.5 mg/mL) was added into the media and equilibrated at 37 °C for 72 h, with shaking. The pH was adjusted to 5.8 or 6.5 and re-equilibrated, if needed. Sample was centrifugated, filtered using a 0.22 µm PVDF filter, and subjected to high-performance liquid chromatography (HPLC) for RTN quantification (below). As a note, the diameter of drug-loaded Soluplus^®^ micelles was around 60–70 nm and easily filtered through 0.2 µm membrane filters [39,40].

#### 2.4.2. Polymer Inhibition of Drug Precipitation

To measure any stabilization effect of polymer on drug supersaturation, at two pHs, 50 mM maleic acid buffer (pH 5.8 or 6.5) was pre-equilibrated with either 1 mg/mL or 3 mg/mL of polymer for 90 min. A stock solution of RTN (2 mg/mL in 100% DMSO) was added at once (i.e., instantaneously) to obtain a system concentration from 2 to 1000 μg/mL RTN, which contained 0.1% DMSO to 50% DMSO. Total volume was adjusted to 1 mL. In total, 1 µL of RTN stock solution (2 mg/mL) was added to 999 µL of polymer solution to obtain 2 µg/mL. In total, 500 µL of RTN stock solution (2 mg/mL) was added to 500 µL of polymer solution to obtain 1000 µg/mL. Similarly, concentrations were adjusted to 2, 10, 20, 25, 30, 50, 100, 150, 200, 250, 300, 400, 500, 600, 800, and 1000 µg/mL with dilutions. Then, the sample was withdrawn after 10 s to avoid any precipitation in the process of sample preparation and centrifuged (15,800 g for 1 min) [41]. The supernatant (100 μL) was diluted with 900 μL of mobile phase [47% acetonitrile: 53% phosphoric acid (0.05 M)] and analyzed via HPLC. Additionally, as a reference, the stock solution of RTN in DMSO was added to buffer (pH 5.8 or 6.5) media without polymer. Dissolved RTN concentration was measured via HPLC after the centrifugation of samples at 15,800 g to remove the precipitate and to determine only free drug, drug/polymer colloids, and micelles [41,42].

Additionally, the viscosity of each polymer solution to prepare films was measured using a Discovery HR20 Rheometer (TA Instruments, New Castle, DE, USA). The shear stress formed under different shear rate conditions (10–100 s^−1^) on the solutions was quantified. Moreover, 40 mm parallel plates (i.e., steel Peltier plates) were used. The temperature was set to 25 ± 2 °C. Constant viscosity values of the polymer solutions were measured at 25 s^−1^ (1500 rpm). Measurements were performed in triplicate.

### 2.5. Film Preparation and Characterization

#### 2.5.1. Film Casting

Preliminary studies were conducted and identified a 2:1 (*w*/*w*) mixture of dichloromethane and methanol as the organic solvent for film casting. Briefly, to obtain viable organic solvent for film casting studies, acetone, dichloromethane (DCM), methanol, or ethanol were used to obtain a 10% *w*/*w* total solid content, with 10%, 20%, and 40% drug loads and 90%, 80%, and 60% polymer loads, respectively. Additionally, combinations of two solvents in 2:1, 1:1, and 1:2 *w*/*w* fractions were also evaluated as solvent systems. A 2:1 (*w*/*w*) mixture of dichloromethane and methanol was selected since all systems provided a transparent appearance.

Using a 2:1 (*w*/*w*) mixture of dichloromethane and methanol, RTN films were prepared using seven different polymer systems: PEG-6000, PVP-VA, Soluplus, Eudragit S100, HPMCAS-H, HPMCAS-L, and a 1:1 combination (i.e., equal weight) of HPMCAS-L/HPMCAS-H. The total solid content (0.5 g of RTN and polymer) of solutions (5 g) was 10% *w*/*w*, which aided films in having the same thickness. Polymer was firstly dissolved in 4.5 g of a 2:1 (*w*/*w*) mixture of dichloromethane and methanol. RTN was added to these polymer solutions to target 20% or 40% drug load.

The solution (5 g) was poured into an aluminum dish. Solvent was evaporated at RT (25 ± 2 °C) for 45 min. Film on dish was then placed into a desiccating cabinet (RH < 5%) for 24 h. After 24 h, the dried films were triturated and milled into the flakes [13]. Triturated films were examined for physical appearance and miscibility. Then, films were subjected to polarized light microscopy and DSC analysis on the day of preparation. Solubilization capacity and in vitro dissolution of films were also performed.

#### 2.5.2. DSC Analysis of RTN Powder, Individual Polymers, and Films

DSC analysis used a Discovery DSC 2500 (TA Instruments, New Castle, DE, USA). RTN powder (i.e., crystalline RTN) or film (5–10 mg) was sealed in an aluminum Tzero pan. For RTN powder, to evaporate possible water content, the heat/cool/heat method was applied under the nitrogen gas flow (50 mL/min). Any melting point was observed on the first heating cycle to 200 °C at 10 °C/min. After the first heating, the system was cooled to 20 °C at 10 °C/min and then reheated to 200 °C at 10 °C/min. T_g_ and melting temperature were recorded from the secondary heating cycle. T_g_ values of RTN were accepted as 52.43 °C [43]. Melting points (e.g., of RTN powder and films) and T_g_ (e.g., of films) were inspected for crystalline or amorphous state, interaction between the drug and polymer, and stability of drug–polymer system.

#### 2.5.3. Polarized Light Microscopy Analysis of Films

Polarized light microscopy (PLM) was performed using a Nikon Eclipse ME600 polarized light microscope (Nikon Instruments Inc., Melville, NY, USA) to assess crystalline state of films. RTN powder (crystalline), polymers, and films were examined at 10× and 40× magnifications. Birefringence was interpreted as crystalline content.

#### 2.5.4. Solubilization Capacity of Films

For films, mini-dissolution was performed. In total, 12.5 mg of film [containing either 2.5 mg (20% drug load) or 5 mg (40% drug load) RTN] was incubated at 37 °C for 1 h in 1.5 mL of dissolution medium (i.e., maleic acid buffer containing 60 mM POE10 at pH 5.8). Also, 2.5 mg or 5 mg of RTN powder (crystalline) without polymer was also assessed as comparisons. Sample was filtered via 0.22 PVDF membrane filter. The amount of RTN solubilized was quantified using HPLC.

#### 2.5.5. Physical Stability of Films

RTN films in open glass scintillation vials were stored at 40 °C/75% RH and 25 °C/60% RH for 30 days. After 30 days, films were evaluated for physical stability (e.g., phase separation or drug crystallization) by DSC. To determine the percentage of crystalline RTN in samples, DSC was calibrated using a known amount (e.g., 5–10 mg) of simple physical mixtures of RTN and HPMCAS-L and focused on the RTN melting point and T_g_. RTN standards used 100%, 75%, 50%, 35%, 25%, 10%, 5%, 2.5%, 1%, or 0% *w*/*w*. Samples and standards were sealed in an aluminum Tzero pan. The heat/cool/heat method was applied under the nitrogen gas flow (50 mL/min). The melting point was observed on the first heating process to 200 °C at 10 °C/min. The calibration curve was obtained by using the enthalpy of the DSC thermogram, and the minimum determined level of crystalline RTN was recorded [13].

#### 2.5.6. Quantification Method of Ritonavir by HPLC

Samples were analyzed by Waters 2489 HPLC system (Waters Corporation, Milford, MA, USA), using a UV–vis detector. The isocratic mobile phase was a 47% acetonitrile and 53% phosphoric acid (0.05 M) solution, with a 25.0 μL injection volume and a flow rate of 1 mL/min. A 4.6 × 150 mm Zorbax C18 5-μm HPLC column was used. The UV–vis detector was set to 240 nm [44,45]. The retention time of RTN was 9–10 min, with a 13 min run time. A calibration curve contained 50 μg/mL, 25 μg/mL, 12.5 μg/mL, 6.25 μg/mL, 3.125 μg/mL, 1.56 μg/mL, 0.78 μg/mL, 0.39 μg/mL, 0.195 μg/mL, and 0.098 μg/mL RTN (r^2^ = 0.9999). For drug solubility in polymer solution studies and for solubilization capacity studies, HPLC RTN standards were obtained by diluting a stock solution of RTN in methanol (1 mg/mL) in a 1:9 ratio with media; the resulting standards for each media were diluted with mobile phase to yield 0.78, 1.56, 3.125, 6.25, 12.5, 25 and 50 μg/mL concentrations.

#### 2.5.7. Dissolution Studies of RTN Films

In vitro dissolution on RTN films (and Norvir tablets) used the USP II apparatus (SR8PLUS, Hanson Research, Chatsworth, CA, USA), in triplicate. Medium was 900 mL of 50 mM maleic acid buffer with 60 mM POE10 (pH 5.8) at 37 °C at 100 rpm for 6 h. Two mL of sample was taken with replacement at 0, 5, 10, 20, 30, 45, 60, 90, 120, 180, 240, and 360 min. Sample was filtered through a 0.45 mm Millipore filter. RTN was quantified using HPLC, as described above. Dissolution profiles were plotted (mean ± SEM). Error bars were not shown when too small to be plotted. Dissolution profiles of films were compared to Norvir tablets using *f_2_* as follows:

$$f_{2}=50\times \log\frac{100}{\sqrt{1+\frac{1}{n}\sum_{t=1}^{n}{\left(R_{t}-T_{t}\right)}^{2}}}$$

where *n* is the number of time points used to evaluate the amount of drug dissolved, and *R_t_* and *T_t_* are the average percentages of drug dissolved at a “*t*” specific time from reference and test products, respectively. Similar dissolution profiles were concluded when *f_*2*_* > 50 [46,47].

### 2.6. Statistical Analysis

ANOVA with the 0.05 level of significance (*p* ≤ 0.05) was assessed via SPSS database Version 16 (Systat Software Inc., San Jose, CA, USA). Meanwhile, the *t*-test was used to compare two groups, and a comparison variance analysis ANOVA test followed by Tukey’s post hoc testing was used to compare multiple groups. Results are given as mean ± standard error of mean (SEM) (n = 3).

## 3. Results and Discussion

### 3.1. Summary of Individual Test Results and Overall Findings

Prior to describing individual screening studies, individual test results are summarized, and overall screening findings are provided. Table 2 indicates overall screening findings. From screening studies here, HPMCAS-L was found to be suitable for subsequent evaluation for both 20% and 40% drug loads, along with the positive control PVP-VA.

Table 3 lists findings from the nine screening test methods, which spanned theoretical calculations and small-scale experimental testing of films. While the Hansen solubility parameters and the Flory–Huggins interaction parameter were helpful for miscibility prediction, ΔT_g_ calculation was more helpful for molecular mobility and the stability of film formulations with various polymers. Test methods considered solid- and/or solution-state performance. Findings sometimes identify polymers to be viable or non-viable. Other times, screening provided a rank-order preference of polymers. From both solid-state and solution-state studies, PEG-6000 was found to be not viable. HPMCAS-L and the positive control PVP-VA were always viable for both 20% and 40% drug loads, across all tests.

### 3.2. Total Hansen Solubility Parameter and Flory–Huggins Interaction Parameter

Table 4 lists δ_T_ and χ values. RTN δ_T_ was 22.81 MPa^0.5^. The lowest and highest polymer δ_T_ was Soluplus’s 19.41 MPa^0.5^ and PEG-6000’s 35.35 MPa^0.5^. δ_T_ of Soluplus agreed with the literature [48]. δ_T_ of HPMCAS-L and HPMCAS-H polymers were similar to one another (~19.91 MPa^0.5^), although lower than in the literature (i.e., 26.11 and 26.18 for HPMCAS-H and HPMCAS-L, respectively) [49]. δ_T_ of Eudragit S100 (19.80 MPa^0.5^) and PVP-VA (19.59 MPa^0.5^) were in agreement with the median from the literature (20.56 MPa^0.5^ for Eudragit S100 and 21.82 MPa^0.5^ for PVP-VA), which employed various methods [50]. Sawant et al. also reported a Eudragit S100 δ_T_ value of 21.1 MPa^0.5^ [51].

Δδ is the difference between RTN and polymer solubility parameters. Drug miscibility and stability in the polymer matrix are related to drug solubility in polymers. Blends are not expected to be miscible when absolute Δδ is greater than 10 mPa^0.5^ [16,52,53]. For a miscible system, absolute Δδ should be lower than 7 mPa^0.5^ [14]. The lowest absolute Δδ was 2.90 MPa^0.5^ for both HPMCAS-L and HPMCAS-H. The highest absolute Δδ was 12.54 MPa^0.5^ for PEG-6000. All drug–polymer pairs had absolute Δδ less than 7 mPa^0.5^, except PEG-6000, such that all drug–polymer pairs except PEG-6000 are expected to be miscible with RTN.

The lowest χ values were for the two HPMCAS grades, where χ = 1.98. χ approaches zero when the solubility parameters of RTN and polymer are similar. A low χ value reflects greater interaction between RTN and a polymer by means of smaller enthalpy of mixing and higher free energy, favoring the mixing.

From Table 4, the miscibility and interaction parameters of RTN–polymer pairs showed rank-order preference: HPMCAS-L = HPMCAS-H > Eudragit S100 > PVP-VA > Soluplus > PEG-6000. Additionally, PEG-6000 often exhibits a crystalline form, resulting in a non-homogenous distribution of the formulation component, causing separate drug-rich and polymer-rich domains. Drug-rich domains have a tendency to result in drug recrystallization and lead to physical instability [54,55].

In this study, drug–polymer affinity was determined in early-stage screening using Hansen solubility parameters and Flory–Huggins Interaction parameters, based on group contributions from various functional moieties in the drug molecule and polymer unit. The primary mechanism of drug–polymer interaction in ASDs is hydrogen bonding between nucleophilic centers (e.g., drug amines) with the polymer’s hydroxyl, carbonyl, and carboxyl groups. Hydrogen bonding frequently plays a role in slowing drug crystallization in an ASD. δ_h_ is the energy from hydrogen bonds between molecules, δ_p_ is the energy from dipolar intermolecular forces between molecules, and δ_d_ is the energy from dispersion forces between molecules. δ_T_ for all polymers was evaluated. For example, PEG 6000 had a higher δ_p_, which caused a higher δ_T_ and higher Δδ (>10 MPa^0.5^). Hence, PEG 6000 was considered as immiscible with ritonavir.

### 3.3. Predicted T_g_ of Amorphous Solid Dispersions

Table 5 lists predicted T_g_ of RTN, polymers, and ASDs for each 20% and 40% drug loads, using the Fox equation. Predicted T_g_ values of ASDs showed rank order: Eudragit S100 > HPMCAS-L = HPMCAS-H = HPMCAS-L:H > PVP-VA > Soluplus, for both 20% and 40% drug loads.

T_g_ is an intrinsic property of amorphous material and is often used to estimate physical stability. T_g_ is related to amorphous and crystalline state transformation. Hence, it affects molecular mobility [56]. When T_g_ values of drug and polymer are close (i.e., absolute ΔT_g_ is low), more drug–polymer interaction is expected. T_g_ is impacted by π-π stacking and hydrogen-bonding interactions. Hydrogen bonding has even been devised to modulate these properties of polymers [57]. For example, Kothari et al. evaluated the molecular mobilities of nifedipine ASDs from PVP (polyvinylpyrrolidone), polyacrylic acid (PAA), and HPMCAS. Drug–polymer hydrogen bond strength, structural relaxation times, and physical stabilities all decreased in the order of PVP > HPMCAS > PAA, indicating that drug–polymer interactions impacted molecular mobility in ASDs [58]. Ueda et al. evaluated that Eudragit R L, HPMCAS, and PVP/VA decrease felodipine crystallization in an ASD. Solid-state 13 C spin-lattice relaxation time was used to assess ASD drug molecular mobility. Drug molecular mobility was declined in the order of PVP/VA > HPMCAS > Eudragit [59].

Polymer ability to inhibit recrystallization during storage is related to polymer T_g_. Higher ASD T_g_ can reduce drug molecular mobility at normally encountered storage temperatures and relative humidities [18,60,61]. Higher T_g_ generally provides higher system stability. In the Fox equation, higher ASD T_g_ can be obtained with higher polymer T_g_.

However, the highest predicted T_g_ for Eudragit S100 also anticipated insufficient drug–polymer interaction due to high absolute ΔT_g_. For both 20% and 40% drug loads, ΔT_g_ of Soluplus, PVP-VA, HPMCAS-L, and HPMCAS-H was each significantly lower (*p* < 0.05) than that of Eudragit S100, indicating their better RTN–polymer interactions than those of Eudragit S100. For films containing 40% RTN, there was no significant difference (*p* > 0.05) between Soluplus, PVP-VA, HPMCAS-L, HPMCAS-H, and HPMCAS-L:H combination (1:1).

Overall, T_g_ predictions indicate all polymers except Eudragit S100 and PEG-6000 as potentially viable for RTN ASDs. The storage of formulations at a temperature greater than ASD T_g_ can result in molecular-mobility-induced phase separation and recrystallization of the drug [62]. The “T_g_-50 °C” rule has been used to predict stability and to determine storage conditions of ASDs. This rule suggests ASD storage at 50 °C below the ASD T_g_ value in order to provide long-term stability [16,53,62,63]. From Table 5, predicted ASD T_g_ values suggest ASD storage between 10 and 50 °C, depending on the polymer.

Figure S1 shows DSC profiles of RTN and individual polymers. RTN T_g_ was 52.43 °C, in agreement with the literature [64,65]. The T_g_ values of polymers were between 69 and 156 °C. Table 5 also compares predicted and observed T_g_ values. There was excellent agreement, with no significant difference (*p* > 0.05) between predicted and observed values. PVP-VA and Soluplus had a lower T_g_ value compared to that of the other polymers. So, a lower ΔT_g_ value was observed, which can be related to the lactam-based polymers having a hygroscobic nature that provides high molecular mobility.

### 3.4. Drug Solubility in Polymer Solution

RTN solubility was quantified at each pH 5.8 and 6.5 media, in the presence and absence of polymer. The solubility medium was a maleic acid buffer with 60 mM POE10, as the USP compendial method for RTN dissolution employs this surfactant.

Table 6 lists solubility values. RTN is a lowly soluble, weakly basic drug [66]. Without 60 mM POE10 and in the absence of any polymer, RTN solubility was 0.00324 ± 0.00002 mM and 0.003 ± 0.00033 mM at pH 5.8 and 6.5, respectively. With POE10, RTN solubility increased almost 100-fold at each pH, to about 0.3 mM.

Polymers had at most a modest ability to increase RTN solubility. At each pH, HPMCAS-L increased RTN solubility the most, by about 1.5–1.7-fold. HPMCAS-L increased RTN solubility slightly more at the lower pH. All other polymers had either a minimal ability to increase RTN solubility, or even decreased RTN solubility. PVP-VA and Soluplus increased drug solubility at each pH. These results were confirmed by the ΔT_g_ value and total Hansen solubility parameter and Flory Huggins interaction parameter. A survey indicated that drug solubility as high as 30–40% was achievable, where polymers yielding the highest solubility were generally lactam-based polymers, such as PVP, PVPVA, and Soluplus. This observation reflects the strong hydrogen-bond-accepting nature of the lactam (pyrrolidone) group [67]. Also, high solubility required the drug to contain a strong H-bond donor or N-H group, where the greatest interaction was between the drug’s N-H group and PVP’s C=O group [68]. Ritonavir also has a N-H group. Therefore, it is possible that the interactions between ritonavir and PVPVA occurs preferentially in the C=O group of the vinyl acetate [31]. Moreover, Soluplus serves as a surfactant and forms drug-loaded micelles to increase drug solubility [39]. Also, results are in general agreement that that excipients (except HPMC) had no significantly impacted RTN solubility at pH 6.8, or in FaSSGF or FaSSIF [69].

Interestingly, HPMCAS-H decreased RTN solubility 2-fold at pH 6.5, in contrast with grade L’s enhancement of solubility. Compared to HPMCAS-L, HPMCAS-H has a higher ratio of acetyl substitution to succinoyl substitution. Hence, HMPCAS-H is more lipophilic and has less of an ionizable character than HPMCAS-L [13]. In Table 6, the solubility results of the 1:1 mixture of L and H grades were intermediate between solubility results for each grade alone.

### 3.5. Polymer Inhibition of Drug Precipitation

**Figure 2:**
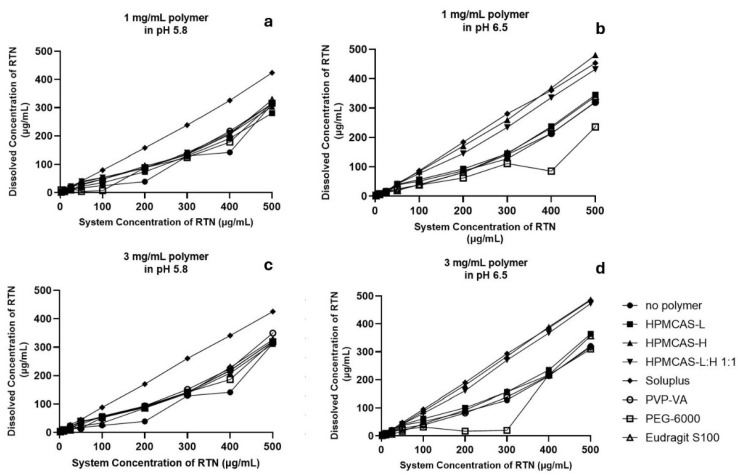
Impact of pre-dissolved polymer on maintenance of RTN in solution. pH was either 5.8 or 6.5. A DMSO stock solution of RTN was added to buffer with 1 or 3 mg/mL of pre-dissolved polymer. No polymer control was also performed. (**a**) 1 mg/mL of pre-solved polymer in pH 5.8; (**b**) 1 mg/mL of pre-solved polymer in pH 6.5; (**c**) 3 mg/mL of pre-solved polymer in pH 5.8; (**d**) 3 mg/mL of pre-solved polymer in pH 6.5. Soluplus generally provided the highest stability.

Figure 2 plots the dissolved RTN concentration versus system RTN concentration after addition of RTN solution into pre-dissolved polymer solutions (1 or 3 mg/mL polymer). Studies were conducted at pH 5.8 and 6.5. Control was also performed with no pre-dissolved polymer. In all four cases (panels a–d), the control profile deviated lower from the line of unity, reflecting some drug precipitation in the absence of polymer.

In general, more RTN (i.e., higher RTN system concentration) results in higher RTN concentration in solutions. However, compared to the control without pre-dissolved polymer, some polymers retarded RTN precipitation, while PEG-6000 promoted drug precipitation. For example, in panel d (HPMCAS-H), a 1:1 mixture of L and H grades and Soluplus each provided profiles that were near the line of unity (i.e., retarding drug precipitation almost completely). Soluplus provided the highest profiles in the other three scenarios, including higher than no polymer control. Soluplus’s ability to solubilize poorly soluble drugs in aqueous media has been attributed to its hydroxyl groups [70,71].

In pH 5.8, from panels a and c, profiles were approximately the same across 1 mg/mL and 3 mg/mL polymer concentrations. Across both pHs, when polymer was 3 mg/mL, the rank order was Soluplus > HPMCAS-L = HPMCAS-H = HPMCAS L:H = PVP-VA > no polymer. In pH 6.5, RTN concentration was lower with PEG-6000 compared to with no polymer.

The performance of polymers in inhibiting drug precipitation did not reflect the polymer effect on viscosity. For example, PVP-VA’s low viscosity was not associated with higher drug precipitation. Briefly, the polymer solution viscosity rank order was as follows: HPMCAS-L = HPMCAS-H = HPMCAS-L:H = Soluplus > Eudragit S100 > PEG-6000 > PVP-VA. Viscosity values of HPMCAS-L (0.152 ± 0.004 Pa·s), HPMCAS-H (0.147 ± 0.005 Pa·s), HPMCAS-L:HPMCAS-H (1:1) (0.151 ± 0.002 Pa·s), and Soluplus (0.141 ± 0.006 Pa·s) solutions were indistinguishable from one another (*p* > 0.05), but they were higher (*p* < 0.05) than those of Eudragit S-100 (0.0427 ± 0.0092 Pa·s), PEG-6000 (0.0194 ± 0.0003 Pa·s), and PVP-VA (0.008 ± 0.0002 Pa·s).

### 3.6. Film Casting and Film Appearance

**Figure 3:**
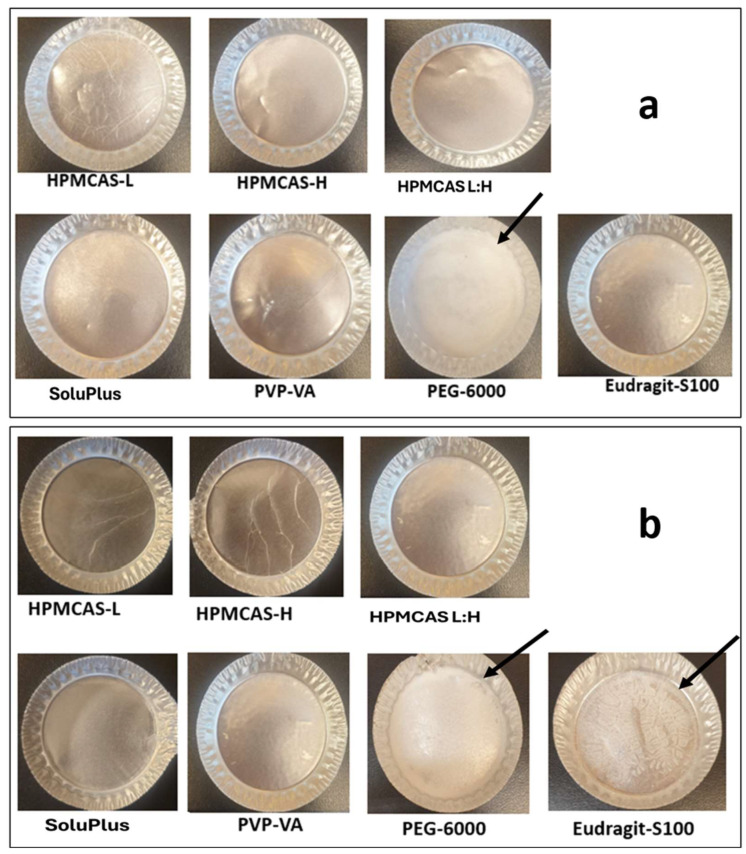
Photographs of (**a**) 20% drug-loaded RTN films containing various polymers. Films were dried in aluminum pans. Black arrow shows the opaque regions in film containing PEG-6000. (**b**) depicts 40% drug-loaded RTN films containing various polymers. Black arrow shows the opaque regions in film containing PEG-6000 and Eudragit S100.

A 2:1 (*w*/*w*) mixture of dichloromethane and methanol was selected, as per above, and yielded RTN films of each PEG-6000, PVP-VA, Soluplus, Eudragit S100, HPMCAS-H, HPMCAS-L, and combination of HPMCAS-L/HPMCAS-H with a 1:1 *w*/*w* ratio. The films were visually evaluated according to opaque or transparent appearance. Transparent means that light passes through the film, and objects behind the film can be distinctly seen. Opaque means not transparent, including the presence of white particulates. Visual evaluation revealed opaque regions in some films, which could be recrystallization of the active substance, the crystalline form of the polymer, or phase separation [13]. In Figure 3 (panel a), all films containing 20% drug load were transparent in appearance, except with PEG-6000. Opaque regions (i.e., with white appearance) of PEG-6000-containing films were due to the crystalline PEG-6000 polymer, as DSC results confirmed.

Figure 3b shows the appearance of films with 40% drug load. Opaque regions were also evident in film containing Eudragit S100. Opaque regions show Eudragit S100-containing film due to RTN crystallization, as PLM DSC results confirmed. Results from film appearance confirm that PEG-6000 is not a suitable polymer for RTN ASDs since RTN and PEG-6000 are not miscible and since, as per the precipitation inhibition studies shown above, PEG-6000 did not provide a parachute effect.

### 3.7. DSC Analysis of Films and Film Physical Stability

Figure S2 plots DSC thermograms of RTN films containing various polymers with 20% and 40% drug loads. Thermograms at the initial time, as well as after stability at 25 °C and 40 °C for one month, are shown. In agreement with Figure 3b, all RTN films except for PEG-6000 were in amorphous drug form, as thermograms showed no RTN melting peak at 128.21 °C (Figure S1). Meanwhile, PEG-6000 showed both an RTN melting peak and the melting of PEG-6000 crystals. DSC thermograms agreed with solubility parameters calculations that RTN films containing polymers except PEG-6000 were miscible systems.

At both 20% and 40% drug loads, DSC thermograms of films containing Soluplus, PVP-VA, HPMCAS-L, HPMCAS-H, and HPMCAS L:H showed a single T_g_, indicating a single amorphous phase. Meanwhile, for Eudragit S100 and PEG-6000, the theoretical assessment (predicted T_g_ values, solubility parameters, and Flory–Huggins interaction parameters) were in agreement with experimental observations. Observed T_g_ values agreed with predicted T_g_ values.

Moreover, the stability of the films was evaluated via DSC analysis. As is shown in Figure S2, films prepared with Soluplus, HPMCAS, and PVP-VA remained amorphous over 30 days. When the RTN drug load increased from 20% to 40%, an RTN crystalline peak was observed for films using PEG-6000, Eudragit S100, and HPMCAS-H, indicating RTN crystallization during storage at 40 °C.

### 3.8. Polarized Light Microscopy Analysis of Films

**Figure 4:**
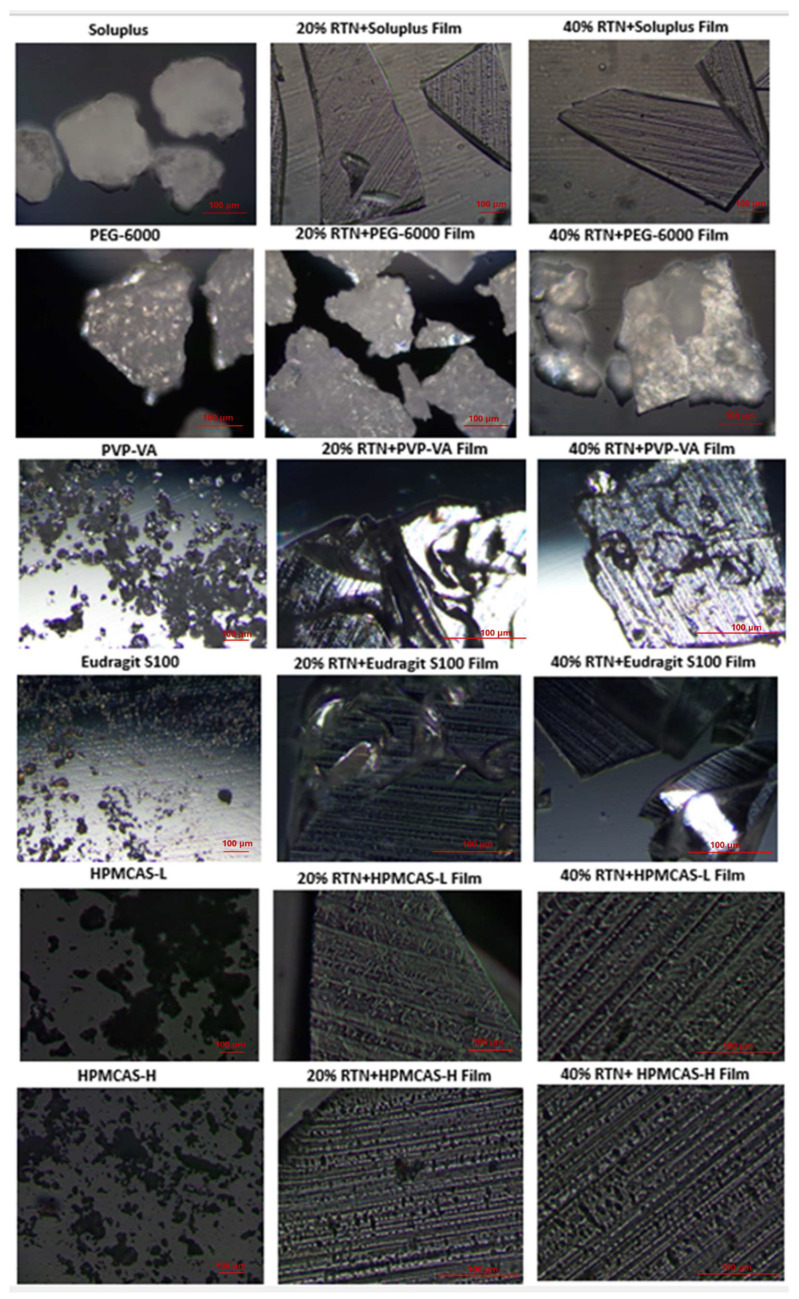
Polarized light microscopy images of polymer and RTN films on initial stability (i.e., prior to storage). Films had either 20% or 40% drug load. Also shown is crystalline RTN.

Triturated films were evaluated on the day of preparation (i.e., initial time) under polarized light for drug crystals. Figure 4 shows PLM photomicrographs of crystalline RTN, each polymer, and each film at 20% and 40% drug loads.

RTN powder showed birefringence due to the RTN crystalline structure. Films containing Soluplus, HPMCAS-L, HPMCAS-H, HPMCAS-L:H, and PVP-VA with 20:80% or 40:60% *w*/*w* of RTN/polymers showed no birefringence and were amorphous. PEG-6000 polymer alone, as well as its films at 20% and 40% drug loads, showed birefringence due to PEG-6000 crystallinity. DSC confirmed this observation, as well as the presence of RTN crystals. PLM images also show birefringence in RTN films of Eudragit S100 at 40% drug load, in agreement with DSC results that indicated the presence of RTN crystals.

For the evaluation of the physical stability of the films, the films were stored at 40 °C/75% RH for 30 days. The films were analyzed by polarized microscopy to evaluate the possible phase separation or drug crystallization on the films. The polarized microscopy images of films containing the containing 20% and 40% drug loads after storage at 40 °C for 30 days are shown in Figure S3. While the amorphous state was still observed on the films prepared with Soluplus, HPMCAS-L, HPMCAS L:H, and PVP-VA, crystalline structures were observed on the PEG-6000, Eudragit S100 and HPMCAS-H polymers at the end of the 30 days at 40 °C.

### 3.9. Solubilization Capacity of Films

**Figure 5:**
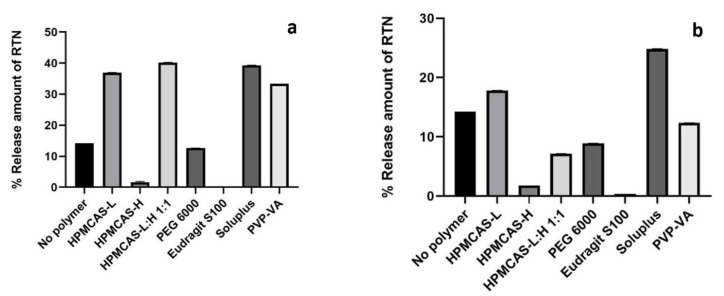
Solubilization capacity of films. Panels show (**a**) capacity from 20% drug load and (**b**) 40% drug load. Values are percent of RTN dissolved after 1 h, out of 2.5 mg RTN (panel (**a**)) or 5 mg RTN (panel (**b**)). Medium was 1.5 mL of 60 mM POE (pH = 5.8).

Figure 5 plots solubilization capacity results for films with 20% (panel a) and 40% drug loads (panel b). The percentage RTN release from film after 1 h was assessed to be the solubilization capacity of the film. The medium was 60 mM POE10 surfactant (pH-5.8). A drug release higher than 30% was considered acceptable [16]. Release was higher for 20% than for 40% for all polymers except HPMCAS-H and Eudragit S100.

In panel a, HPMCAS-L, HPMCAS-L:H, Soluplus, and PVP-VA provided a release of more than 30%. None of the polymers yielded a release of more than 30%. Hence, HPMCAS-L, HPMCAS-L:H, Soluplus, and PVP-VA are preferred polymers for potential RTN ASDs, although a 20% drug load is preferable over a 40% drug load. Considering their collective favorable results, including higher solubilization capacity, Soluplus, HPMCAS-L, HPMCAS L:H, and PVP-VA were selected for comparative dissolution studies against Norvir tablets.

### 3.10. Dissolution Profiles of RTN Films

**Figure 6:**
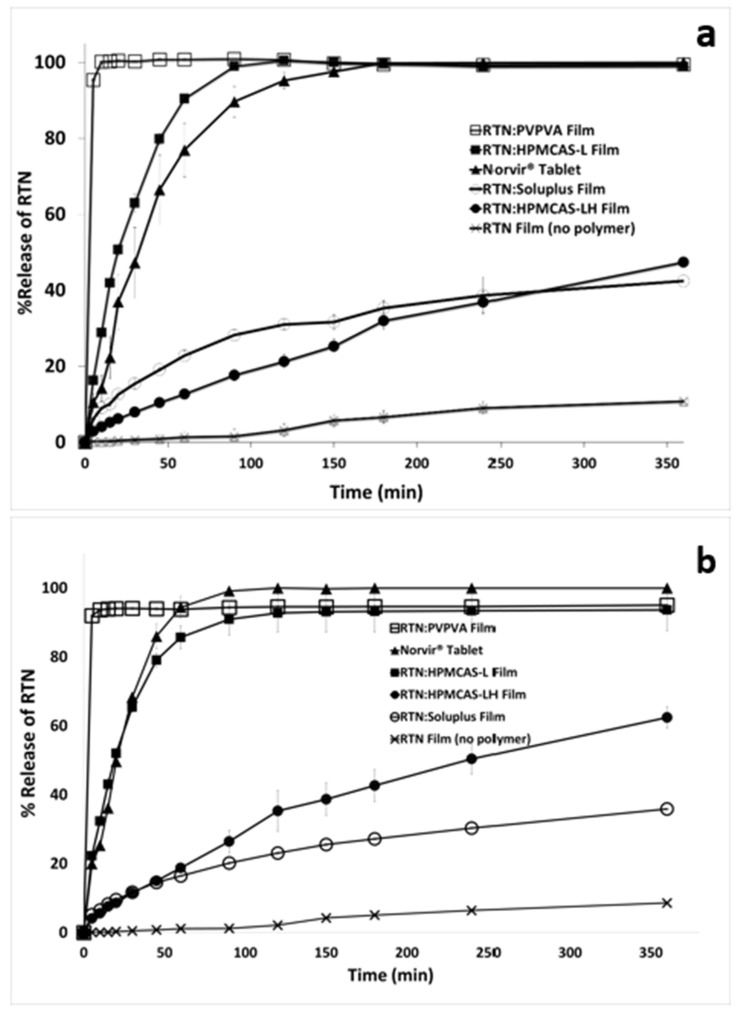
Dissolution profiles of RTN films containing Soluplus, HPMCAS-L, HPMCAS-L:H, or PVP-VA with 20% drug load. Panels show (**a**) dissolution in pH 5.8 and (**b**) dissolution in pH 6.5. Profiles from Norvir tablets and amorphous RTN without polymer are also shown. Data are mean (±SEM) from n = 3.

In vitro drug dissolution is a laboratory test of the drug product to assess product quality. The test measures drug release (i.e., drug dissolution) from the product. The dissolution profiles of the films were determined in a POE10 medium having pH 5.8 and 6.5 due to a USP dissolution database that suggests using 60 mM POE10 for dissolution testing of RTN. Hence, 60 mM POE was used here, similarly to in the literature [44]. In Figure 6, the PVP-VA polymer showed the most rapid release at both pHs. At each pH 5.8 and pH 6.5, PVP-VA yielded about a 10-fold dissolution enhancement compared to that of the no polymer.

At pH 5.8, the rank order was PVP-VA > HPMCAS-L > Norvir > Soluplus > HPMCAS-L:H > no polymer (Figure 6a). At pH 6.5, it was PVP-VA > Norvir > HPMCAS-L > HPMCAS-L:H > Soluplus > no polymer (Figure 6b), reflecting the enteric property of HPMCAS-L and HPMCAS-H.

All films containing polymers showed a better dissolution profile than that of amorphous RTN without polymer. While the percent RTN release with no polymer was 10.8 (±0.1)% in pH 5.8, the RTN release was 42.5 (±4.9)%, 99.0 (±0.08)%, 99.5 (±0.3)%, and 47.4 (±2.8)% for Soluplus, HPMCAS-L, PVP-VA, and HPMCAS-L:H films, respectively, with 20% drug load (Figure 6a). Similarly, the percent RTN release with no polymer was 8.6 (±0.06)% in pH 6.5, and the release was 35.9 (±0.3)%, 93.7 (±6.1)%, 95.1 (±0.3)%, and 62.5 (±3.1)% for Soluplus, HPMCAS-L, PVP-VA, and HPMCAS-L:H films, respectively, with 20% drug load (Figure 6b).

Given the importance of drug dissolution in product quality, there are numerous regulatory guidelines concerning the comparing of in vitro drug dissolution profiles, typically employing the *f_2_* metric. The similarity factor (*f_2_* value) approach is used to assess the similarity of dissolution profiles between test and reference products. A value between 50 and 100 reflects similarity of profiles [47,72,73]. Here, the dissolution profiles of RTN film formulations with various polymers were compared to each other and with the oral marketed tablet formulation Norvir tablets. Norvir tablets are the reference listed drug in the FDA Orange Book [74]. Comparing test dissolution profiles to the reference listed drug is a common approach to assess dissolution profiles of test formulations [75,76,77]. Table 7 lists *f_2_* values in comparing film dissolution profiles to Norvir tablet dissolution. Among the films, HPMCAS-L was the film that was most similar to Norvir tablets. PVP-VA was dissimilar to Norvir since it was more rapidly dissolving, although PVP-VA passed USP specifications which are 75% in 120 min [78].

### 3.11. Overall Assessment of Polymers

In the context of this polymer screening study, there was general agreement in the favorable dissolution performance of the PVP-VA film with Norvir tablets, which employ PVP-VA as their ASD polymer [25]. Likewise, the dissolution of the HPMCAS-L film performed favorably. In Table 2, across all nine tests, PVP-VA and HPMCAS-L were determined as the most suitable polymers to prepare RTN ASDs by this screening process. The positive control PVP-VA was, as expected, found to be suitable, reflecting favorably on the screening approach.

Eudragit S100 and PEG-6000 were not suitable for subsequent consideration. In the polymer inhibition of drug precipitation, PEG-6000 was not viable and Eudragit S100 was not preferable. Neither performed preferably in solubilization capacity or in film dissolution. In film physical appearance and corresponding DSC and PLM tests, Eudragit S100 and PEG-6000 were not viable. In particular, predicted and observed T_g_ anticipated them not being viable. ASD drug–polymer interaction is often mediated via H bonding between nucleophilic centers (e.g., drug amines) with polymer hydroxyl, carbonyl and carboxyl groups. Hydrogen bonding frequently plays a role in slowing drug crystallization in an ASD. δ_h_ is the energy from hydrogen bonds between molecules, δ_p_ is the energy from dipolar intermolecular forces between molecules, and δ_d_ is the energy from dispersion forces between molecules. δ_T_ for all polymers was evaluated with the total Hansen solubility parameter and Flory–Huggins interaction parameter. These parameters each anticipated that PEG-6000 was not viable. HPMCAS-H, HPMCAS-L:H, and Soluplus were not as preferred as HPMCAS-L or PVP-VA, but they were suitable for further consideration for 20% drug load but not 40% drug load. For example, none was more preferable than HPMCAS-L in drug solubility in the polymer solution test or in the dissolution test; moreover, HPMCAS-H was not viable at 40% drug load as per the DSC test and not preferable as per the solubilization capacity. Hence, PVP-VA and HPMCAS-L were determined from this resource-sparing screening approach as suitable polymers for subsequent ritonavir ASD development, such as SDDs. A drug must often contain a strong H-bond donor or N-H group for high solubility to be observed. Relatedly, drug–polymer interactions have involved the N-H group of the drug and the C=O group of PVP [25]. Ritonavir also has the N-H group. Therefore, it is possible that the interactions between ritonavir and PVPVA occur preferentially in the C=O group of vinyl acetate [31]. The favorable performance here of PVP-VA may reflect this interaction.

## 4. Conclusions

A main challenge in ASD development is the selection of a viable drug–polymer pairs and drug loads. While there does not appear to be a unified approach to select a viable drug–polymer pair and drug load, several laboratories have described approaches to screen polymers in ASD development. For example, Simoes et al. developed a fast, effective, and material-sparing method to design an etravirine ASD [16], and Maddineni et al. investigated the effects of processing variables and formulation factors on the characteristics of an ASD containing Kollidon^®^VA 64 and nifedipine [79].

Here, the objective was to screen polymers for potential ASDs of ritonavir via drug–polymer interaction calculations and small-scale experimental characterization. Seven polymers of differing physicochemical properties were assessed, including PVP-VA as a positive control, since it is the ASD polymer in commercial Norvir tablets. Nine screening test methods were applied though three layers of methods: theoretical calculations, experimental assessment of drug–polymer physical mixtures and solutions, and film characterization. That is, theoretical calculations of solid-state drug–polymer interactions were performed, as ASD miscibility favors when drug and polymer have similar solubilities parameters and similar T_g_. Calculations included δ_T_, χ, and T_g_. Drug–polymer interaction calculations anticipated the unfavorable small-scale experimental results for PEG-6000 and Eudragit S100. While Hansen solubility parameters and the Flory–Huggins interaction parameter were helpful for miscibility prediction, ΔTg calculation was more helpful for the molecular mobility and stability of film formulations with various polymers.

Then, solid-state and solution-state drug–polymer studies were conducted, focusing on the ability of polymers to promote drug solubility and the inhibition of drug precipitation. Each polymer was found to be viable in the drug solubility in polymer solution, although HPMCAS-L was identified as the most preferred, in general agreement with subsequent in vitro dissolution testing. Polymer inhibition of drug precipitation found PEG-6000 as not viable and Eudragit S100 as less preferred, in agreement with several other test results.

Next, films were fabricated via film casting and tested using PLM, DSC, solubilization capacity, and dissolution studies. HPMCAS-L, HPMCAS L:H, and Soluplus, along with the positive control PVP-VA, were identified as polymers for potential ASDs of ritonavir, with HPMCAS-L and PVP-VA being preferable. Films with each of these four polymers showed improved dissolution compared to that of amorphous ritonavir without polymers. Overall, the results contribute towards a resource-sparing approach to identify polymers for ASDs.

## Figures and Tables

**Table 1:** Polymer properties. Polymers with a range of properties were evaluated. A 1:1 mixture of HPMCAS-L and HPMCAS-H was also evaluated.

	PVP-VA	PEG-6000	Soluplus	Eudragit S100	HPMCAS-L	HPMCAS-H
Polymer Type	Amphiphilic Polymer	Hydrophilic Polymers	Polymer + Surfactant Combination (Copolymer)	Enteric Polymer	Enteric + Amphiphilic Polymer	Enteric + Amphiphilic Polymer
Molecular Weight (kDa)	7.3–9.3	50–75	118	125	114.7	75.1

**Table 2:** Final screening assessment of polymers. HPMCAS-L with 20% and 40% drug load was suitable for subsequent evaluation. The positive control PVP-VA was, as expected, found to be suitable, reflecting favorably on the screening approach.

Polymer	Final Screening Assessment
HPMCAS-L and PVP-VA	Suitable for 20% and 40% drug load
HPMCAS-H, HPMCAS-L:H, and Soluplus	Suitable for 20% drug load but not 40% drug load
Eudragit S100 and PEG-6000	Not suitable

**Table 3:** Table of tests and test results. Test results of individual polymers were in terms of preferred polymer, viable polymer, and non-viable polymer, regarding subsequent polymer section to fabricate SDDs.

Test Method	Relevance	Finding
Total Hansen solubility parameter and Flory–Huggins interaction parameter	Solid-state (TA *)	All polymers except PEG-6000 were viable.
Predicted and observed ΔT_g_	Solid-state (TA * and DSC analysis)	All polymers except Eudragit S100 and PEG-6000 were viable.
Drug solubility in polymer solution	Solution-state (EA * of crystalline drug)	All polymers were viable. HPMCAS-L was most preferred.
Polymer inhibition of drug precipitation	Solution-state (EA * of DMSO solubilized drug)	PEG-6000 was not viable. Eudragit S100 was least preferred compared to other viable polymers.
Film physical appearance	Solid-state (EA * of films)	All polymers at 20% DL, except PEG-6000, were viable. At 40% DL, Soluplus, HPMCAS-L, PVP-VA, HPMCAS-H, and HPMCAS L:H were viable, while Eudragit S100 was not viable.
DSC analysis of films	Solid-state (EA * of films)	On initial stability, Soluplus, PVP-VA, HPMCAS-L, HPMCAS-H, and HPMCAS L:H were viable at 20% and 40% DLs, while Eudragit S100 and PEG-6000 were not viable at either drug load. After 30 days at 40 °C, Soluplus, HPMCAS-L, HPMCAS-H, HPMCAS L:H, and PVP-VA were viable at 20% DL. After 30 days at 40 °C, Soluplus, HPMCAS-L, HPMCAS L:H, and PVP-VA were viable at 40% DL, while HPMCAS-H was not viable.
Polarized Light Microscopy Analysis of Films	Solid-state (EA * of films)	On initial stability, Soluplus, PVP-VA, HPMCAS-L, HPMCAS-H and HPMCAS L:H were viable at 20% and 40% DL, while Eudragit S100 and PEG-6000 were not viable. After 30 days at 40 °C, Soluplus, HPMCAS-L, HPMCAS-H, HPMCAS L:H, and PVP-VA were viable at 20% DL. After 30 days at 40 °C, Soluplus, HPMCAS-L, HPMCAS L:H, and PVP-VA were viable at 40% DL, while HPMCAS-H was not viable.
Solubilization capacity of films (i.e., mini-dissolution)	Solid- and solution-state (EA * of films)	HPMCAS-L, HPMCAS-L:H, Soluplus, and PVP-VA were preferred, although 20% DL was preferable over 40% DL.
Dissolution studies of films	Solid- and solution-state (EA * of films)	HPMCAS-L and PVP-VA were preferred.

* TA: theoretical assessment; EA: experimental assessment; DL: drug load.

**Table 4:** Predicted drug–polymer miscibility. Miscibility was estimated from difference between drug and polymer total solubility parameter (i.e., Δδ), as well as Flory–Huggins interaction parameter.

Compound/ Polymer	Total Solubility Parameter (δ_T_) MPa^0.5^	Δδ = [δ_RTN_ − δ_POL_] MPa^0.5^	Flory–Huggins Interaction Parameter (Χ)	Drug–Polymer Compatibility
RTN	22.81	-	-	-
Soluplus	19.41	3.40	2.71	Miscible
PVP-VA	19.59	3.22	2.44	Miscible
Eudragit S100	19.80	3.01	2.13	Miscible
PEG 6000	35.35	−12.54	36.94	Immiscible
HPMCAS-L and H	~19.91	2.90	1.98	Miscible

**Table 5:** Observed and predicted T_g_ of RTN–polymer ASDs. Drug load was 20% or 40%. Predicted T_g_ employed Fox equation. PEG-6000 was not evaluated due to its crystalline structure.

			20% RTN	40% RTN	20% RTN	40% RTN
Film Components	T_g_ (°C)	ΔT_g_ (°C)= T_g RTN_ − T_g Pol_	T_g_ (°C) Predicted	T_g_ (°C) Predicted	T_g_ (°C) Observed	T_g_ (°C) Observed
Ritonavir	52.43	-	-	-		
Soluplus	69.74	−17.31	65.42	61.60	64.64	62.50
PVP-VA	108.06	−55.63	89.14	75.86	89.64	61.83
Eudragit S100	156.46	−104.03	112.01	87.23	124.17	106.76
HPMCAS-L	123.56	−71.13	97.19	80.10	91.45	74.23
HPMCAS-H	124.24	−71.81	97.52	80.27	99.65	74.82
HPMCAS-L:H 1:1	123.9	−71.47	97.36	80.18	99.55	74.64

**Table 6:** Impact of polymer on RTN solubility. pH was either 5.8 or 6.5. All media employed 60 mM POE 10 media. RTN solubility in buffer alone without POE10 was 0.00324(±0.00002) mM in pH 5.8 and 0.00300(±0.00033) mM at pH 6.5.

Media (pH = 5.8)	Solubility (mM)	Media (pH = 6.5)	Solubility (mM)
Media without polymer	0.283(±0.003)	Media without polymer	0.270(±0.003)
HPMCAS-L	0.504(±0.039)	HPMCAS-L	0.454(±0.031)
HPMCAS-H	0.337(±0.008)	HPMCAS-H	0.159(±0.005)
HPMCAS-L:H (1:1)	0.358(±0.006)	HPMCAS-L:H (1:1)	0.213(±0.004)
Soluplus	0.380(±0.028)	Soluplus	0.327(±0.052)
PVP-VA	0.318(±0.026)	PVP-VA	0.350(±0.038)
PEG-6000	0.314(±0.023)	PEG-6000	0.344(±0.012)
Eudragit S100	0.209(±0.022)	Eudragit S100	0.285(±0.003)

**Table 7:** *f_*2*_* of films compared to Norvir tablets. Media were 60 mM POE10 (pH 5.8) and POE10 (pH 6.5).

Formulation	*f_*2*_* (pH 5.8)	*f_*2*_* (pH 6.5)
PVP-VA film	16.1	22.7
HPMCAS-L film	48.0	59.4
Soluplus film	15.8	10.5
HPMCAS-H:L film	13.5	13.5
No polymer	7.7	5.3

## Data Availability

Data will be made available on request.

## Supplementary Materials

The following supporting information can be downloaded at: https://www.mdpi.com/article/10.3390/pharmaceutics16111373/s1, Figure S1: DSC profiles of film of RTN-polymer (20% drug load), as well as unformulated RTN active pharmaceutical ingredient; Figure S2: DSC thermogram of the RTN films containing various polymers. Panels show (a) Soluplus, (b) PEG-6000, (c) PVP-VA, (d) Eudragit S100, (e) HPMCAS-L, (f) HPMCAS-H, and (g) HPMCAS L:H. Thermograms show 20% and 40% drug loads, as well as storage condition of initial, 25 °C for 30 days, and 40 °C for 30 days; Figure S3: Polarized light microscopy images of polymer and RTN films with polymer after storage at 40 °C for 30 days. Films had either 20% or 40% drug load.
