# Supplementary material for: Screening of Polymers for Oral Ritonavir Amorphous Solid Dispersions by Film Casting
Source: Pharmaceutics. 2024 Oct 26;16(11):1373. doi: 10.3390/pharmaceutics16111373 (PMC11597764; doi:10.3390/pharmaceutics16111373)
Supplement: Supplementary file 1 [file pharmaceutics-16-01373-s001.zip › pharmaceutics-3236260-supplementary.pdf]

Supplementary material for

Screening of Polymers for Oral Ritonavir Amorphous Solid Dispersions by Film Casting

Ayse Nur Oktay [1,2,*] and James E. Polli [2]

1 Department of Pharmaceutical Technology, Gulhane Faculty of Pharmacy, University of Health Sciences, An-kara 06018, Türkiye

2 Department of Pharmaceutical Sciences, University of Maryland, Baltimore, MD 21201, USA;

jpolli@rx.umaryland.edu

* Correspondence: aysenur.oktay@sbu.edu.tr

Results and Discussion

Prediction of T_g of amorphous solid dispersions

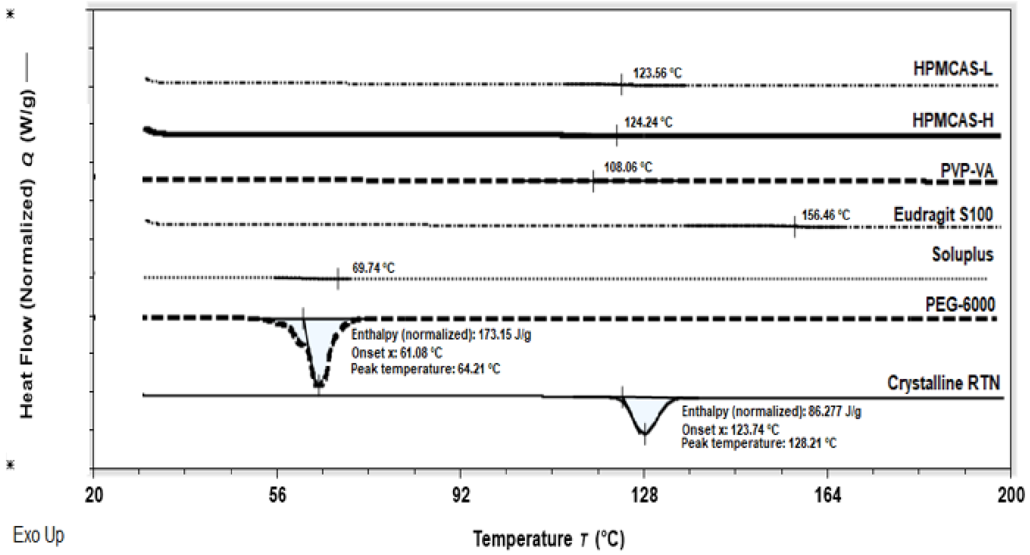

Figure S1. DSC profiles of film of RTN-polymer (20% drug load), as well as unformulated RTN active pharmaceutical ingredient.

DSC Analysis of Films and Film Physical Stability

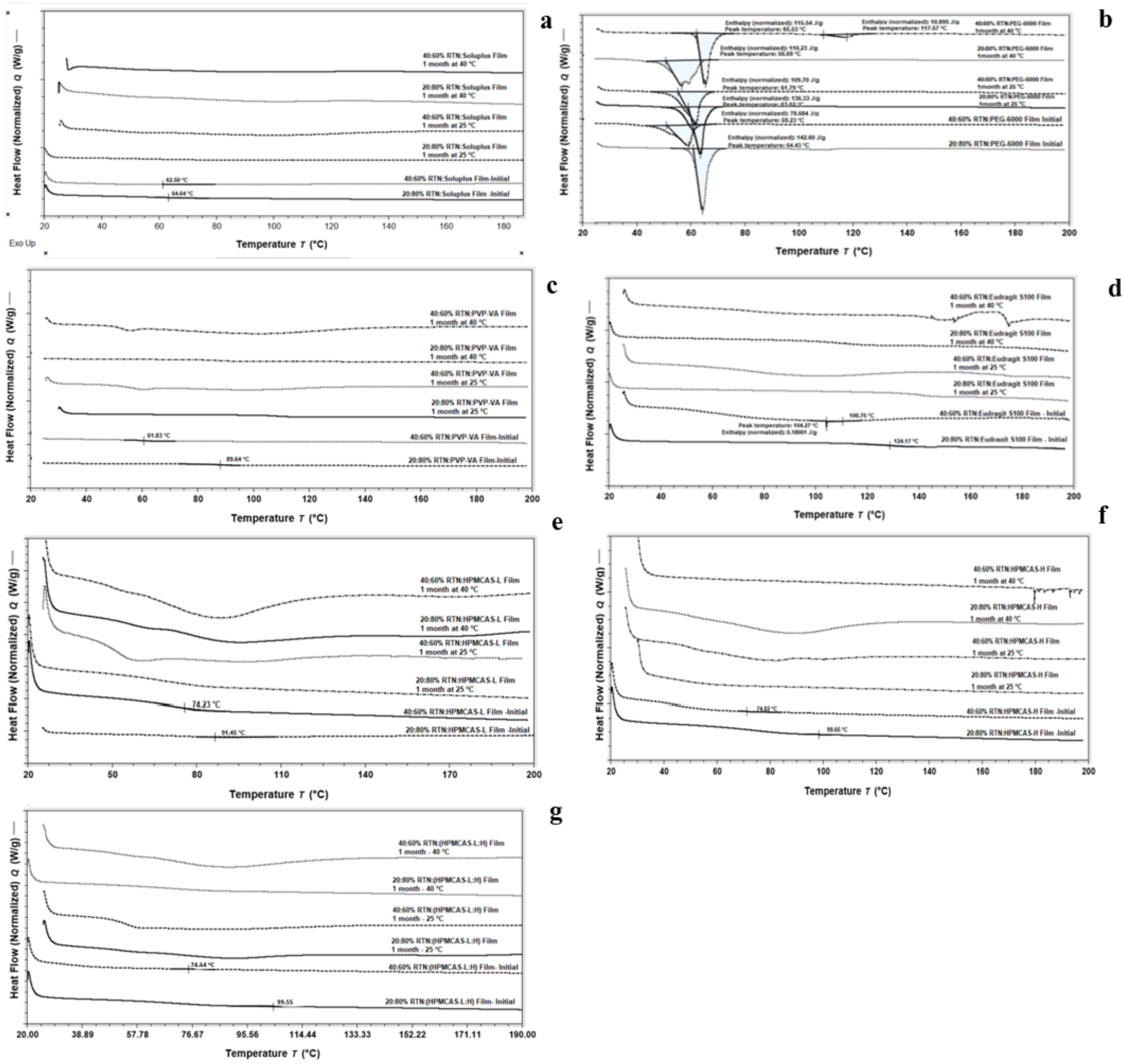

Figure S2. DSC thermogram of the RTN films containing various polymers. Panels show a) SoluPlus, b) PEG-6000, c) PVP-VA, d) Eudragit S100, e) HPMCAS-L, f) HPMCAS-H, and g) HPMCAS L:H. Thermograms show 20% and 40% drug loads, as well as storage condition of initial, 25ºC for 30 days, and 40ºC for 30 days.

Polarize Light Microscopy Analysis of Films

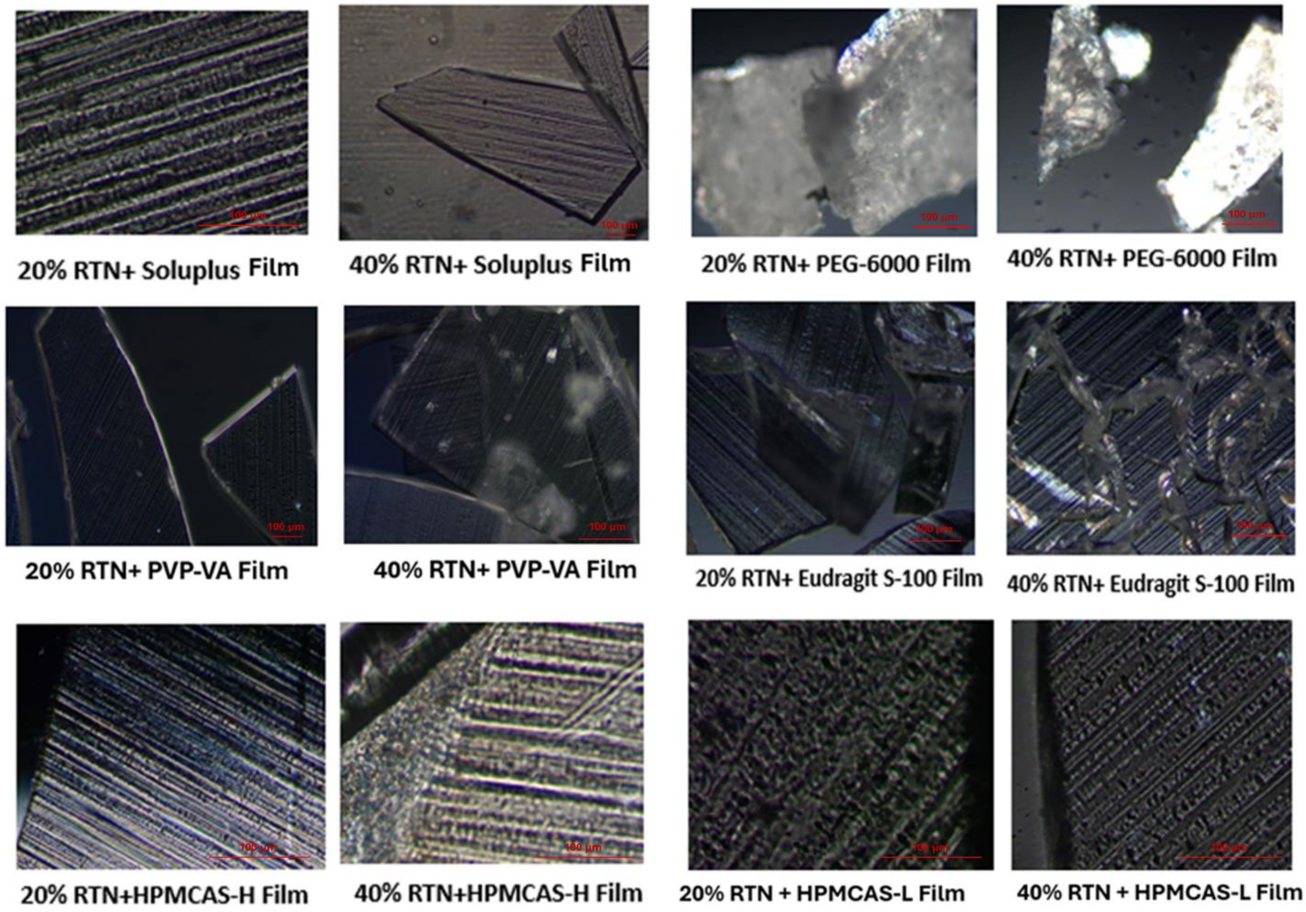

Figure S3. Polarized light microscopy images of polymer and RTN films with polymer after storage at 40ºC for 30 days. Films had either 20% or 40% drug load.
